# Scoping review of medical students' perceptions of the field of dermatology

**DOI:** 10.1002/ski2.171

**Published:** 2022-12-30

**Authors:** Rewan Abdelwahab, Ahmad Shahin, Yong‐hun Kim, Jennifer Coias, Muhammad B. Qureshi, Nahid Vidal

**Affiliations:** ^1^ Mayo Clinic Alix School of Medicine Rochester Minnesota USA; ^2^ Mayo Clinic Alix School of Medicine Scottsdale Arizona USA; ^3^ Division of Dermatologic Surgery Department of Dermatology Mayo Clinic Rochester Minnesota USA

## Abstract

**Background:**

Although perceived barriers to applying to dermatology have been researched among medical students, there remains a dearth of literature dedicated to understanding perceptions that medical students have of the field of dermatology and dermatologists.

**Methods:**

A review of the literature in Embase, Pubmed, Scopus, Web of Science, and ScienceDirect were carried out to identify articles and abstracts between 2016 and 2021 relating to medical student perceptions of the field of dermatology. Peer‐reviewed English studies measuring attitudes/level of interest in dermatology or other specialities, understanding of dermatologic topics, procedures, and/or scope of practice were included. Duplicate studies and conference abstracts were excluded. All publications were screened using the PRISMA‐Sc guidelines. Findings were summarised and tabulated accordingly.

**Results:**

A total of nine articles met inclusion criteria and eight are included in this review since one was not accessible online. Notable findings include non‐US medical students perceiving dermatology as monotonous, stigmatized, unfamiliar, and difficult to access with a misunderstanding of the diversity and severity of the conditions dermatologists treat. No data on US medical student perceptions was found. Perceptions were found to be influential in career planning: medical students may reject specialities after exposure to negative comments on the field. Factors attracting students to dermatology include the appeal of being a dermatologist, media portrayal, and dermatologists' influence on patients' lives. Completion of dermatology‐related activities improved medical student interest, comfort, and understanding of the field. Early dermatology exposure in US undergraduate premedical students led to heightened interest in the field, more confidence in ability to find dermatology mentors, and increased perception that dermatology serves the needs of underserved communities.

**Conclusions:**

This review demonstrates the need to further investigate medical student perceptions of dermatology, particularly in the United States. Perceptions of medical specialities can impact medical student career choices. Understanding which misconceptions may be preventing students from exploring dermatology can inform efforts towards improving diversity, equity, and inclusion: translating to an equitable match and improving patient outcomes. Limitations include exclusion of articles published before 2016, geographic variability in studies, and limited data on evolving student perceptions over time.

1

**What is already known about this topic?**

- There is published work about the perceptions of dermatology that are held by the public, medical programme directors, and non‐U.S. medical students. There is also literature on the impact such perceptions of a speciality can have on student career choices. We did not find any studies specific to the U.S. medical student population regarding perceptions of the field of dermatology that may dissuade qualified applicants from applying into the field.

**What does this study add?**

- This review consolidates the existing literature around medical students' understanding and perception of the field of dermatology. This can help in developing targeted curricula and dermatology outreach and pipeline programs to better inform participants about dermatology. It may also help programs become more effective in reaching students that may otherwise overlook dermatology as a field.

## 2 INTRODUCTION

Brezinski et al. found that the general public in United States felt that cardiology and primary care were ‘more critical professions’ when compared to dermatology.^1^ The general public also overestimated the proportion of time dermatologists spend on cosmetic procedures when compared to reported workforce data.^2^ Misconceptions on the role of dermatologists also extend to the medical field. Few programme medical directors of the 21 medical specialities surveyed ascribed the treatment of mucosal, nail, and hair conditions to dermatologists,^3^ suggesting that, the perception that dermatology has a limited scope of practice may persist. On the other hand, collaboration and interaction with dermatologists prompted physicians from other medical fields to adopt more favourable perceptions of dermatologists.^3^ This suggests that increased familiarity with dermatology scope of practice may counteract misperceptions and stigmas surrounding the field.

Perceptions of the field of dermatology can impact health policy decisions, allocation of research funds, and community education campaigns.^4^ By formalizing methods of collaboration between primary care physicians and dermatologists, physicians can better address the dermatologic disease burden in their patient populations and develop effective community education campaigns. To meet this ideal, there is a fundamental need to increase the public and medical professional knowledge on the field of dermatology. However, as of yet, we could not find any study evaluating U.S. medical students' perceptions of the field of dermatology. Some studies, however, have assessed medical students' perceptions of the field of dermatology in countries in Europe,^5^, ^6^ Africa,^7^ and the Middle East.^8^ A survey of medical students across four Californian institutions demonstrated that medical students' dermatology clerkship experiences were predominantly positive.^9^ Nevertheless, the particular perceptions and traits associated with dermatology and dermatologists that derive from the media or other forms of representation have not been delineated in the medical student population. Given the limited nature of existing literature on this topic and the research group's primary goal of characterising these published works, a scoping review format was selected for this article.

### 2.1 Group hypothesis

Medical students are not adequately exposed to dermatology during medical school leading to misconceptions about the field. This may deter otherwise qualified applicants and potentially decrease the diversity of the applicant pool applying to a dermatology residency. Increased exposure to dermatology in medical school helps students formulate more accurate perceptions and may ultimately influence their decision on pursuing it as a speciality.

## 3 METHODS

This review aimed to identify medical student perceptions of dermatologists and the field of dermatology. The databases Embase, Pubmed, Scopus, Web of Science, and ScienceDirect were searched for English articles and abstracts within the past 5 years (2016–2021) relating to the perceptions of medical students regarding dermatology, including factors such as skin colour, socioeconomics, diversity, and experiences with dermatologic conditions. The suggested keywords on the library literature search form were ‘dermatology’, ‘medical school’, ‘diversity’, ‘perceptions of dermatology/dermatologist’, and ‘medical student’.

Each study's title, abstract, and full text was reviewed among three reviewers independently to establish if inclusion criteria was met. Studies were included if they met the following criteria: (1) the study was extracted from the Embase, Pubmed, Scopus, Web of Science, or ScienceDirect databases; (2) the study participants were medical students and/or students interested in the field of dermatology; (3) the study measured at least one of the following variables: attitudes/level of interest in dermatology or other specialities, understanding of dermatologic topics, procedures, and/or scope of practice; (4) the study was in English; (5) the study was published in peer‐reviewed journal; (6) the publication date was between 2016 and 2021; (7) all study designs were considered.

A total of seven articles were included in this review and subject to data charting in excel. The data charting included the following information: author, title, publication year, methodology country of origin, study populations, sample size, limitations, survey administration, and key findings.

The key findings of papers were classified as perceptions of dermatologists and the field of dermatology, stereotypes, perceived scope of practice and patient impact, student interventions that changes perceptions of the field, and intervention that showed no change in the perception of the field of dermatology.

Studies were excluded based on the following criteria: (1) duplicate studies; (2) conference abstracts; (3) studies that did not meet each of the criteria 1–7 of the inclusion criteria. Reporting Guidelines: Draft written in accordance with the PRIMSA‐Sc guidelines.

## 4 RESULTS

**FIGURE 1:**
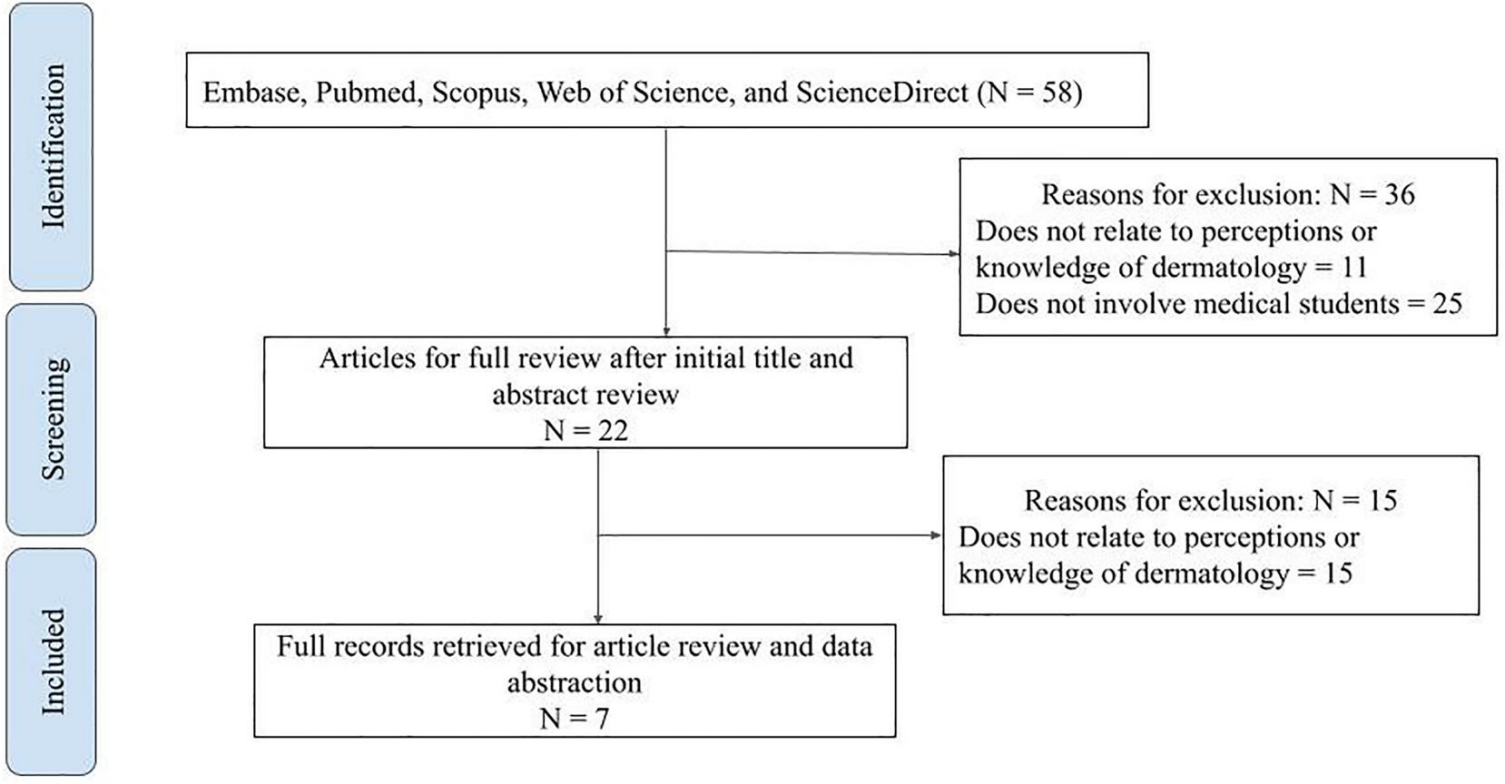
Flow chart detailing inclusion and exclusion criteria

A total of 58 citations were identified through the aforementioned electronic search engines. A screening process based on titles and abstracts eliminated 50 articles that were not applicable to our research question. One of the articles could not be accessed online. The remaining seven articles met the eligibility criteria and were included in this literature review (Figure 1). The studies reviewed covered perceptions of various aspects of dermatology including access to the speciality, expertise of dermatologists, and the impact of dermatological conditions on patients. The impact of such perceptions on medical students' career choices was also discussed in the reviewed works. An overview of included articles is provided in Tables 1 and 2.

**TABLE 1:** Summary of articles included for review

First author, year, location	Study method	Sample size	Available demographics	Relevant findings regarding perceptions
Ajaz et al. 2016. United Kingdom	Quantitative survey	*n* = 960	36% male, age (25.8% 18–20, 70.1% 21–29, 3.5% 30–39, 0.5% > 40 years), (15.2% first, 13.6% second, 15.1% third, 26.5% fourth, 23.6% fifth)	
Cervantes et al. 2019. United States	Quantitative survey	*n* = 29	All first‐ or second‐year students (distribution not provided)	33.3% stated computer‐based video instruction increased their interest in dermatology
Fenton et al. 2020. United States^24^ ^a^	Quantitative survey	*n* = 177	First‐ through fourth‐year medical students	
Guckian et al. 2019. United Kingdom	Mixed methods survey	*n* = 16		94% reported increased interest in dermatology after dermatology‐based escape room game
Ludriksone et al. 2018. Germany	Quantitative survey	*n* = 182	100% fourth‐year students	10.6% pre‐versus 10.72% post‐module agreed dermatology has a high status among other physicians 12.2% pre‐versus 18.3% post‐module agreed dermatologists are exceptionally competent compared to other physicians
Onyekaba et al. 2021. United States	Quantitative survey	*n* = 12	11 undergraduate, 1 post‐baccalaureate, 83.3% Black, 16.7% Hispanic, mean age = 19	25% pre‐versus 75% post‐event interest in dermatology 66% pre‐versus 100% post‐event belief they could find a mentor in dermatology
Alajmi et al. 2020. Saudi Arabia	Quantitative survey	*n* = 121	62.8% fifth year, 37.2% sixth year, 76% male, 97.5% unmarried	6.6% preference for dermatology Important factors in choosing dermatology: appeal of being a dermatologist, media portrayal, likelihood of influencing a patient's life
Teclessou et al. 2019. Togo	Quantitative survey	*n* = 176	38.8% seventh year, 31.1% sixth year, 30.1% fifth year	37.2% interested in dermatology 79.6% view dermatology as speciality of intermediate difficulty

^a^ Fenton et al. included for completeness as it met our inclusion criteria but was not included in the results due to its irrelevance to our discussion.

**TABLE 2:** Characteristics of articles included for review

Country of origin	Saudi Arabia (*n* = 1)
	Togo (*n* = 1)
	Germany (*n* = 1)
	United Kingdom (*n* = 2)
	United States (*n* = 3)
Aggregate sample size	1661 medical students
	12 premedical students
Male‐to‐female student ratio	438:643^a^
Mean age of respondents	24^b^
Survey design	Quantitative survey (*n* = 5)
	Mixed‐method survey (*n* = 1)

^a^ Data derived from Ajaz et. Al and Alajmi et al.

^b^ Data derived from Ajaz et. Al and Onyekaba et al.

### 4.1 Perceptions of dermatologists & field of dermatology

In a survey of medical students in Saudi Arabia, 7% of respondents favoured dermatology as a speciality.^8^ The most attractive factors of the field of dermatology were the appeal of being a dermatologist, media portrayal of different specialities, and the likelihood of dermatologists' influence on patients' lives.^8^ A survey of medical students at the University of Lomé, Togo similarly demonstrated few students with interest in dermatology.^7^

The majority of Germans have consulted a dermatologist in the past, and yet dermatology consistently ranked low in terms of prestige and importance of the speciality.^6^ British medical students described a lack of familiarity with the field and described dermatology as hard to access.^5^

#### 4.1.1 Stereotypes

A UK survey of medical students also found dermatology to be one of the most common specialities receiving negative comments regarding the nature of the work and personal qualities of dermatologists, along with psychiatry and general practice.^10^ The negative stereotypes surrounding dermatology primarily described dermatologists as lazy while the stereotypes regarding psychiatry and general practice questioned the physician's competence.^10^

Ajaz et al. reported from their survey data that some students rejected certain specialities when they encountered negative comments, whether they related to the personal characteristics of physicians in that speciality or the nature of the work itself.^10^ Others feared discrimination by their peers or evaluators and kept their career choice to themselves.^10^

#### 4.1.2 Scope of practice and patient impact

German medical students perceive dermatologists to have high expertise in skin conditions, skin cancer, atopic dermatitis and psoriasis, and assume dermatologists have little knowledge on mucous membrane disease and male infertility.^6^ Skin disease was perceived to be frequent, mentally burdensome and disfiguring.^6^

On the other hand, most German medical students categorize dermatologic conditions as harmless on the whole.^6^ When compared to the general German population's perception of dermatology, German medical students perceive the breadth of dermatologic practice to be larger whereas British medical students describe the field of dermatology as monotonous and ‘mostly eczema and psoriasis’.^5^, ^6^

### 4.2 Intervention impact on perceptions of dermatology

#### 4.2.1 Changed perception

After a one‐time dermatology escape room session, students felt the breadth of dermatology was greater than they had thought prior.^5^ The escape room learning format incorporated team‐based puzzle solving to progress through a series at tasks in a designated time period. All tasks incorporated dermatologic knowledge or skills. The British medical students also felt more comfortable with the field.^5^ Among German medical students, the perceived incidence of dermatologic conditions increased after taking a dermatology module.^6^ Within the US, a study evaluating the efficacy of computer‐based video instruction using modules from the American Academy of Dermatology (AAD) demonstrated greater knowledge retention and preparation for hands‐on shave and punch biopsy training, with a third of participating students indicating this experience increased their interest in dermatology.^11^ Similarly, completion of dermatology internship improved student interest in the field.^7^

The two‐hour event hosted for underrepresented in medicine premedical students at the University of Pennsylvania was found to increase both interest in dermatology and the perceived ability to find dermatology mentors. After the session, there was also increased perception that dermatology serves the needs of underserved communities.^12^

#### 4.2.2 Unchanged perception

The 12‐week dermatology training module had no impact on the perception of the importance of dermatology among German medical students and no impact on interest in the field of dermatology.^6^

## 5 DISCUSSION

Our scoping review results demonstrate that across countries, few students demonstrated interest in the field of dermatology. Favourable perceptions of the field held by medical students were based on media portrayal of the field.^8^ On the other hand, experiences receiving care from dermatologists as a patient did not appear to positively impact perception of the field.^6^ For students expressing interest in the field of dermatology, one of the factors drawing students to the field was the impact that dermatologists have on their patients' lives.^8^ Many of the students that indicated dermatology as a hard to access field also demonstrated holding negative perceptions of dermatologists and the field more generally.^5^, ^10^ This indicates a possible area of intervention where increased exposure to scope of dermatological practice may be effective at combating the negative stereotypes that medical students hold of the field.^5^, ^6^ Students with more exposure to the field of dermatology in the past more accurately described the breath of dermatologic practice.^5^ The most effective medical student interventions that increased interest in the field include active, face‐to‐face, or hands‐on learning such as escape rooms, events with current practicing dermatologists, and active computer‐based video instruction.^5^, ^7^, ^11^ More passive computer modules were found to be ineffective in disseminating dermatologic knowledge and increasing interest.^6^

The AAD highlights a need to increase underrepresented in medicine students applying to medical school, increase exposure and interest in dermatology, and increase recruitment of underrepresented students into dermatology programs.^13^ Increased diversity is shown to be associated with improved clinical outcomes such as improved patient satisfaction and compliance as well as reduced clinical uncertainty regarding diagnosis and treatment options.^14^ Currently, only 16% of United States medical schools dedicate a preclinical course to dermatology, and most do not require dermatology clinical rotations.^15^ Yet, mentorship and pipeline programs have been found to be crucial to a successful dermatology match.^16^

Barriers to applying to dermatology noted by students at a German medical school include difficulty of matching into dermatology, a high research demand for matching, and belief that they were not smart enough.^5^ Similar barriers to applying to a dermatology residency were reported at a US medical school including high step exams, high clinical grades, and risk of not matching.^17^, ^18^ Students from lower socioeconomic backgrounds and ethnic minorities also cited lack of diversity in dermatology, socioeconomic barriers, and negative perceptions of minority students as a significant barrier to application.^17^

Anti‐racism plans that have been outlined to help combat these perceived barriers to applying include addressing microaggressions, discussing racism, prioritising equitable representation of skin of colour in journals and textbooks, emphasizing mentorship, building inclusion committees for dermatology, and increasing dermatology service among others.^19^, ^20^ Media and social media are other outlets through which increased interest and diversity acceptance can be pushed in the field of dermatology.^19^

However, very little research has been dedicated to understanding the perceptions that medical students have on the field of dermatology. Furthermore, the research that has been conducted is often qualitative and difficult to generalize, with no validated measuring outcome tool to grade and compare results. Perceptions may also have been influenced by confounding variables including but not limited to country of origin. Future investigation using a standardized protocol for the evaluation of perceptions of dermatology would offer important insight while allowing data to be compared and applied more effectively.

One such application is the development of targeted curricula and dermatology outreach and pipeline programs to better inform participants on the field of dermatology. It may also help programs become more effective in reaching students that may otherwise overlook dermatology as a field.

Bridging the gap between medical student misconceptions and the reality of dermatology is especially important when some students may already be dissuaded from pursuing the field due to lack of a home programme. While one third of dermatologists match at their home institution,^21^ 25% of medical students lack a home dermatology programme.^22^, ^23^ This disadvantage also manifests before match day as increased difficulty of developing dermatologic clinical skills before audition rotations, obtaining academic letters of recommendation, and securing research experiences.

## 6 LIMITATIONS

This literature review should be considered in the light of some limitations. First, the reviewed studies and the medical students they surveyed demonstrated heterogeneity with respect to country of origin, with regions including North America, Europe, Africa, and the Middle East. This variation introduces confounding variables that make generalisability difficult, though the dearth of information available in any one region emphasises the need for further investigation.

Second, this review may have been limited by excluding articles published prior to 2016. While this was done to incorporate only up‐to‐date information, this may have contributed to the limited yield of search results.

Finally, while the review helps us understand the current perceptions of medical students in the field of dermatology, the findings do not yet indicate whether these perceptions persist or evolve over time. Thus, the research team cannot establish a causal relationship between improved medical student perceptions of dermatology and a positive impact on the field.

## 7 CONCLUSION

Although perceived barriers to applying to dermatology have been researched among medical students, there remains a dearth of literature dedicated to understanding medical students' perceptions of the field of dermatology and dermatologists. We found no studies devoted to this topic in the United States. To promote an equitable match and to bolster ongoing diversity, equity, and inclusion recruitment and retention initiatives in the field, further investigation is needed to understand what misconceptions or perceptions may be preventing students from enrolling in dermatology pipeline programs. Understanding this knowledge can bolster recruitment of a more diverse pool of students into dermatology programs, improve public perception of the field, and grow funding for dermatology health initiatives.

## CONFLICT OF INTEREST

The author declares that there is no conflict of interest that could be perceived as prejudicing the impartiality of the research reported.

## AUTHOR CONTRIBUTIONS

**Rewan Abdelwahab**: Conceptualization (equal); Investigation (equal); Project administration (equal); Writing – original draft (equal); Writing – review & editing (equal). **Ahmad Shahin**: Investigation (equal); Writing – original draft (equal); Writing – review & editing (equal). **Yong‐hun Kim**: Investigation (equal); Writing – original draft (equal); Writing – review & editing (equal). **Jennifer Coias**: Conceptualization (equal); Methodology (equal); Writing – review & editing (equal). **Muhammad B. Qureshi**: Visualisation (equal); Writing – original draft (equal); Writing – review & editing (equal). **Nahid Vidal**: Conceptualization (equal); Methodology (equal); Supervision (equal); Writing – review & editing (equal).

## ETHICS STATEMENT

Not applicable.

## Data Availability

Data sharing is not applicable to this article as no new data were created or analyzed in this study.
